# 2-Hy­droxy­benzenaminium acetate

**DOI:** 10.1107/S2414314622001122

**Published:** 2022-02-03

**Authors:** Nabila Moussa Slimane, Nesrine Benarous, Hassiba Bougueria, Aouatef Cherouana

**Affiliations:** a Unité de Recherche de Chimie de l’Environnement et Moléculaire Structurale (CHEMS), Département de Chimie, Université des Frères Mentouri, Constantine-1, 25017 Constantine, Algeria; b Centre Universitaire Abd El Hafid Boussouf, Mila, 43000 Mila, Algeria; Katholieke Universiteit Leuven, Belgium

**Keywords:** crystal structure, 2-hy­droxy­benzenaminium cation, acetate anion, hydrogen bond

## Abstract

In the title molecular salt, C_6_H_8_NO^+^·C_2_H_3_O_2_^−^, the cations and anions are linked by O—H⋯O and N—H⋯O hydrogen bonds, generating a three-dimensional network.

## Abstract 2

In the crystal, the mol­ecules are linked by O—H⋯O and N—H⋯O hydrogen bonds into a three-dimensional network.

## Graphical abstract

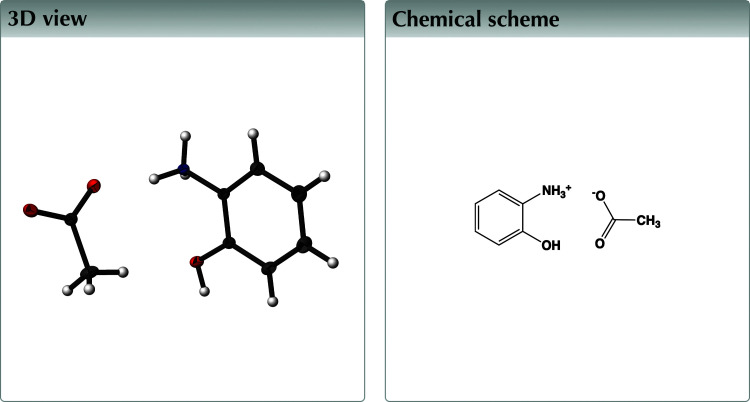

## Structure description

In recent years, substituted anilines and their derivatives have been studied extensively for applications as anti­bacterials and in non-linear optical systems (Vivek & Murugakoothan, 2014 ▸). Amino­phenols containing equal stoichiometries of –OH, and –NH_2_ groups have been widely studied to understand the supra­molecular synthons existing in their assemblies (Allen *et al.*, 1997 ▸; Dey *et al.*, 2004 ▸).

In spite of this inter­est, there has been very little structural characterization of *ortho*-hy­droxy­anilinium salts. The structures reported include 2-hy­droxy­anilinium squarate (Yeşilel, 2007 ▸), 2-hy­droxy­anilinium hydrogen phthalate (Jagan & Sivakumar, 2009 ▸), 2-hy­droxy­anilinium 3,5-di­nitro­salicylate (Smith *et al.*, 2011 ▸), 2-hy­droxy­anilinium 3,5-di­nitro­benzoate (Zhao, 2012 ▸), and 2-hy­droxy­anilinium 2-hy­droxy-5-nitro­benzoate and 2-hy­droxy­anilinium 3,5-di­nitro­benzoate (Jin & Wang, 2013 ▸).

**Figure 1:**
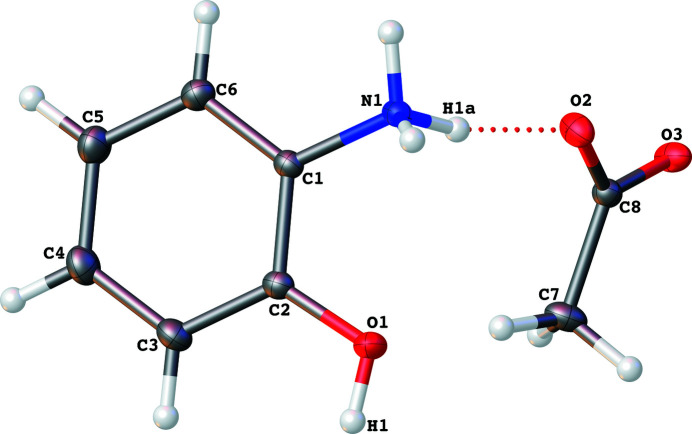
Diagram showing the C_6_H_8_NO^+^ cation and C_2_H_3_O_2_^−^ anion linked by an N—H⋯O inter­action (shown as a dashed line). Displacement ellipsoids are drawn at the 50% probability level.

Here, we report the structure of 2-hy­droxy­benzenaminium acetate, C_6_H_8_NO^+^·C_2_H_3_O_2_^−^, **1**, obtained from the reaction of 2-hy­droxy­aniline and acetic acid. The mol­ecular structure of the title compound is shown in Fig.1. The asymmetric unit contains one 2-hy­droxy­benzenaminium cation and one acetate anion. The cation is protonated at the amine N atom (N1) and linked to the anion by an N—H⋯O hydrogen bond (Fig. 1 ▸ and Table 1 ▸).

The best planes through the 2-hy­droxy­benzenaminium cation and acetate anion are almost perpendicular to each other, subtending a dihedral angle of 79.23 (4)°. The C—OH bond length (C2—O1) of 1.3520 (9) Å is similar to that observed for structures containing 2-hy­droxy­benzenaminium as a cation [1.350 (3) Å; Jin & Wang, 2013 ▸]. All bond lengths and angles in the 2-hy­droxy­benzenaminium cation are within normal ranges (Zhao, 2012 ▸).

**Figure 2:**
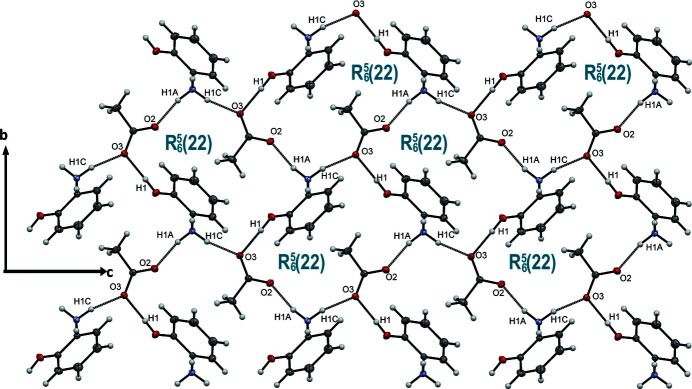
A portion of one corrugated layer viewed along the *b*-axis direction. O—H\⋯O and N—H⋯O hydrogen bonds are shown as dashed lines.

**Figure 3:**
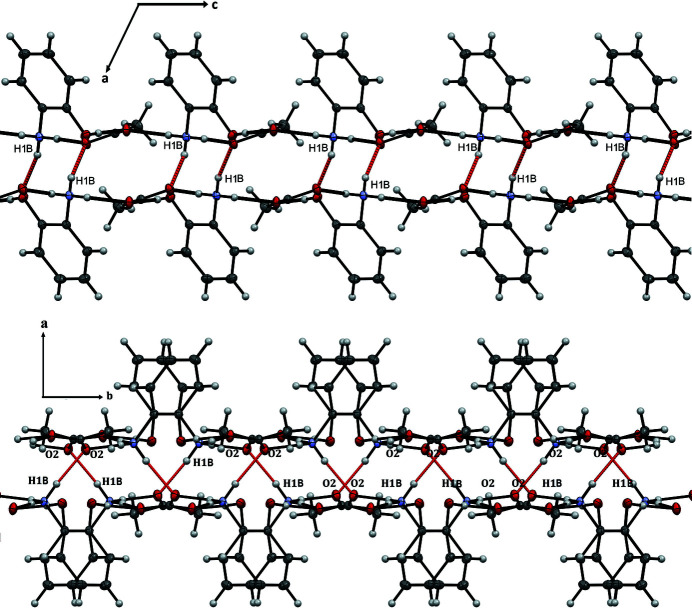
View of two layers viewed along the *b*- and *c*-axis directions.

The presence of hydroxyl groups leads to the formation of inter­molecular O1—H1⋯O3 hydrogen bonds. The O1—H1⋯O3 and N1—H1*C*⋯O3 cation–anion hydrogen bonds generate a succession of infinite chains [graph set 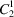 (7)] that propagate in a zigzag manner along the *c-*axis direction (Fig. 2 ▸ and Table 1 ▸). The N1—H1*A*⋯O2 hydrogen bonds (Table 1 ▸) link the chains into corrugated layers parallel to the *bc* plane, which are formed by a succession of 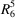 (22) rings (Fig. 2 ▸). N1—H1*B*⋯O2 hydrogen bonds lead to the formation of a three-dimensional network (Fig. 3 ▸). No significant π–π stacking inter­actions were observed, despite the presence of an aromatic ring in the cation.

## Synthesis and crystallization

The title compound was prepared by heating of a mixture of 2-amino­phenol (Alfa Aesar, purity 98%) and acetic acid. This mixture was obtained by dissolution and agitation under reflux for 3 h of 0.5 g of the 2-amino­phenol and 0.27 g of acetic acid in a 1:1 stoichiometric ratio in a hot ethano­lic solution (20 ml). After warming for a few minutes using a water bath, the solution was cooled and kept at room temperature. Within a few days, yellow needle-like crystals suitable for the X-ray analysis were obtained (yield 60%) by evaporation of the solution.

## Refinement

Crystal data, data collection and structure refinement details are summarized in Table 2 ▸.

## Supplementary Material

Crystal structure: contains datablock(s) I. DOI: 10.1107/S2414314622001122/vm4050sup1.cif

Structure factors: contains datablock(s) I. DOI: 10.1107/S2414314622001122/vm4050Isup3.hkl

Supporting information file. DOI: 10.1107/S2414314622001122/vm4050Isup3.cml

CCDC reference: 2149479

Additional supporting information:  crystallographic information; 3D view; checkCIF report

## Figures and Tables

**Table 1:** Hydrogen-bond geometry (Å, °)

*D*—H⋯*A*	*D*—H	H⋯*A*	*D*⋯*A*	*D*—H⋯*A*
O1—H1⋯O3^i^	0.894 (14)	1.709 (14)	2.6025 (9)	177.8 (16)
N1—H1*A*⋯O2	0.930 (13)	1.807 (13)	2.7251 (9)	168.7 (12)
N1—H1*B*⋯O2^ii^	0.923 (12)	1.891 (12)	2.8019 (9)	168.8 (11)
N1—H1*C*⋯O3^iii^	0.935 (12)	1.834 (12)	2.7531 (8)	167.2 (12)
C6—H6⋯O3^iii^	0.95	2.55	3.2493 (11)	131

Symmetry codes: (i) 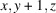 ; (ii) 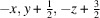 ; (iii) 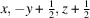 .

**Table 2:** Experimental details

Crystal data	
Chemical formula	C_6_H_8_NO^+^·C_2_H_3_O_2_^−^
*M*_r_	169.18
Crystal system, space group	Monoclinic, *P*2_1_/*c*
Temperature (K)	100
*a*, *b*, *c* (Å)	9.9150 (2), 7.2523 (2), 11.9573 (3)
β (°)	98.558 (2)
*V* (Å^3^)	850.23 (4)
*Z*	4
Radiation type	Mo *K*α
μ (mm^−1^)	0.10
Crystal size (mm)	0.10 × 0.10 × 0.08

Data collection	
Diffractometer	Oxford Diffraction Xcalibur Sapphire2 CCD
Absorption correction	Integration (*ABSORB*; DeTitta, 1985 ▸)
*T*_min_, *T*_max_	0.966, 0.991
No. of measured, independent and observed [*I* > 2σ(*I*)] reflections	52913, 3105, 2736
*R*_int_	0.038
(sin θ/λ)_max_ (Å^−1^)	0.766

Refinement	
*R*[*F* ^2^ > 2σ(*F* ^2^)], *wR*(*F* ^2^), *S*	0.038, 0.109, 1.05
No. of reflections	3105
No. of parameters	122
H-atom treatment	H atoms treated by a mixture of independent and constrained refinement
Δρ_max_, Δρ_min_ (e Å^−3^)	0.49, −0.27

Computer programs: *CrysAlis PRO* (Rigaku OD, 2018 ▸), *SHELXS* (Sheldrick, 2008 ▸), *SHELXL2018/3* (Sheldrick, 2015 ▸) and *OLEX2* (Dolomanov *et al.*, 2009 ▸).

## full crystallographic data

### Crystal data

**Table:**

C_6_H_8_NO^+^·C_2_H_3_O_2_^−^	*F*(000) = 360
*M**_r_* = 169.18	*D*_x_ = 1.322 Mg m^−^^3^
Monoclinic, *P*2_1_/*c*	Mo *K*α radiation, λ = 0.71073 Å
*a* = 9.9150 (2) Å	Cell parameters from 52927 reflections
*b* = 7.2523 (2) Å	θ = 3.3–33.0°
*c* = 11.9573 (3) Å	µ = 0.10 mm^−^^1^
β = 98.558 (2)°	*T* = 100 K
*V* = 850.23 (4) Å^3^	Prism, yellow
*Z* = 4	0.1 × 0.1 × 0.08 mm

### Data collection

**Table:**

Oxford Diffraction Xcalibur Sapphire2 CCD diffractometer	2736 reflections with *I* > 2σ(*I*)
φ and ω scans	*R*_int_ = 0.038
Absorption correction: integration (ABSORB; DeTitta, 1985)	θ_max_ = 33.0°, θ_min_ = 3.3°
*T*_min_ = 0.966, *T*_max_ = 0.991	*h* = −15→15
52913 measured reflections	*k* = −11→11
3105 independent reflections	*l* = −17→18

### Refinement

**Table:**

Refinement on *F*^2^	Primary atom site location: structure-invariant direct methods
Least-squares matrix: full	Hydrogen site location: mixed
*R*[*F*^2^ > 2σ(*F*^2^)] = 0.038	H atoms treated by a mixture of independent and constrained refinement
*wR*(*F*^2^) = 0.109	*w* = 1/[σ^2^(*F*_o_^2^) + (0.0542*P*)^2^ + 0.320*P*] where *P* = (*F*_o_^2^ + 2*F*_c_^2^)/3
*S* = 1.05	(Δ/σ)_max_ = 0.001
3105 reflections	Δρ_max_ = 0.49 e Å^−^^3^
122 parameters	Δρ_min_ = −0.27 e Å^−^^3^
0 restraints	

### Special details

**Table:**

Geometry. All esds (except the esd in the dihedral angle between two l.s. planes) are estimated using the full covariance matrix. The cell esds are taken into account individually in the estimation of esds in distances, angles and torsion angles; correlations between esds in cell parameters are only used when they are defined by crystal symmetry. An approximate (isotropic) treatment of cell esds is used for estimating esds involving l.s. planes.
Refinement. The hydrogen atoms of the NH_3_ and hydroxyl groups were localized in the difference-Fourier map and refined with *U*_iso_(H) set to 1.5*U*_eq_(O) or 1.2*U*_eq_(N). All the other hydrogen atoms were placed in calculated positions with C—H = 0.95 Å for aromatic CH and C—H = 0.96 Å for CH_3_ and refined using a riding model with fixed isotropic displacement parameters [*U*_iso_(H) = 1.2*U*_eq_(C-aromatic) and *U*_iso_(H) = 1.5*U*_eq_(*C*-methyl)].

### Fractional atomic coordinates and isotropic or equivalent isotropic displacement parameters (Å^2^)

**Table:**

	*x*	*y*	*z*	*U*_iso_*/*U*_eq_	
O1	0.12872 (6)	0.83288 (9)	0.65994 (5)	0.01629 (13)	
H1	0.1381 (14)	0.928 (2)	0.6142 (12)	0.024*	
O2	0.09592 (7)	0.29888 (8)	0.65411 (5)	0.01686 (13)	
O3	0.15033 (7)	0.10731 (8)	0.52349 (5)	0.01664 (13)	
N1	0.12133 (7)	0.56570 (9)	0.81635 (5)	0.01242 (13)	
H1A	0.1133 (12)	0.4870 (19)	0.7543 (10)	0.015*	
H1B	0.0461 (12)	0.6407 (18)	0.8160 (10)	0.015*	
H1C	0.1280 (12)	0.4909 (18)	0.8806 (10)	0.015*	
C3	0.35594 (9)	0.93249 (12)	0.74235 (7)	0.01774 (16)	
H3	0.358215	1.025256	0.686567	0.021*	
C5	0.46510 (9)	0.77468 (13)	0.90970 (8)	0.01994 (17)	
H5	0.540747	0.760726	0.967953	0.024*	
C4	0.46648 (9)	0.91021 (13)	0.82754 (8)	0.02042 (17)	
H4	0.543738	0.988327	0.829677	0.025*	
C2	0.24139 (8)	0.81883 (11)	0.73847 (6)	0.01328 (14)	
C6	0.35205 (8)	0.65957 (12)	0.90596 (7)	0.01606 (15)	
H6	0.350402	0.566114	0.961414	0.019*	
C1	0.24197 (8)	0.68208 (10)	0.82091 (6)	0.01217 (14)	
C8	0.13683 (8)	0.26870 (11)	0.56096 (6)	0.01288 (14)	
C7	0.16938 (9)	0.42886 (12)	0.48900 (8)	0.01940 (16)	
H7A	0.096016	0.443787	0.425025	0.029*	
H7B	0.255531	0.405118	0.460661	0.029*	
H7C	0.177717	0.541719	0.534587	0.029*	

### Atomic displacement parameters (Å^2^)

**Table:**

	*U*^11^	*U*^22^	*U*^33^	*U*^12^	*U*^13^	*U*^23^
O1	0.0172 (3)	0.0157 (3)	0.0153 (3)	−0.0014 (2)	0.0001 (2)	0.0046 (2)
O2	0.0220 (3)	0.0142 (3)	0.0157 (3)	−0.0024 (2)	0.0073 (2)	−0.0030 (2)
O3	0.0255 (3)	0.0124 (3)	0.0124 (2)	0.0019 (2)	0.0041 (2)	−0.00051 (19)
N1	0.0147 (3)	0.0109 (3)	0.0120 (3)	−0.0006 (2)	0.0030 (2)	0.0005 (2)
C3	0.0180 (3)	0.0161 (3)	0.0198 (4)	−0.0030 (3)	0.0053 (3)	0.0024 (3)
C5	0.0150 (3)	0.0222 (4)	0.0217 (4)	−0.0007 (3)	−0.0003 (3)	0.0001 (3)
C4	0.0154 (3)	0.0212 (4)	0.0248 (4)	−0.0040 (3)	0.0036 (3)	0.0000 (3)
C2	0.0149 (3)	0.0121 (3)	0.0131 (3)	0.0002 (2)	0.0032 (2)	0.0005 (2)
C6	0.0162 (3)	0.0160 (3)	0.0157 (3)	0.0013 (3)	0.0015 (3)	0.0011 (3)
C1	0.0133 (3)	0.0110 (3)	0.0126 (3)	−0.0001 (2)	0.0033 (2)	0.0000 (2)
C8	0.0128 (3)	0.0123 (3)	0.0135 (3)	0.0001 (2)	0.0019 (2)	0.0007 (2)
C7	0.0213 (4)	0.0154 (3)	0.0229 (4)	0.0008 (3)	0.0078 (3)	0.0063 (3)

### Geometric parameters (Å, º)

**Table:**

O1—H1	0.892 (14)	C5—H5	0.9500
O1—C2	1.3520 (9)	C5—C4	1.3912 (13)
O2—C8	1.2600 (9)	C5—C6	1.3930 (12)
O3—C8	1.2675 (9)	C4—H4	0.9500
N1—H1A	0.930 (13)	C2—C1	1.3978 (11)
N1—H1B	0.922 (13)	C6—H6	0.9500
N1—H1C	0.935 (12)	C6—C1	1.3863 (11)
N1—C1	1.4583 (10)	C8—C7	1.5087 (11)
C3—H3	0.9500	C7—H7A	0.9800
C3—C4	1.3903 (12)	C7—H7B	0.9800
C3—C2	1.3985 (11)	C7—H7C	0.9800
			
C2—O1—H1	109.4 (9)	O1—C2—C1	117.36 (7)
H1A—N1—H1B	112.7 (11)	C1—C2—C3	118.48 (7)
H1A—N1—H1C	106.7 (11)	C5—C6—H6	120.2
H1B—N1—H1C	107.7 (10)	C1—C6—C5	119.66 (8)
C1—N1—H1A	110.9 (8)	C1—C6—H6	120.2
C1—N1—H1B	108.5 (8)	C2—C1—N1	117.84 (7)
C1—N1—H1C	110.3 (7)	C6—C1—N1	120.74 (7)
C4—C3—H3	119.9	C6—C1—C2	121.40 (7)
C4—C3—C2	120.24 (8)	O2—C8—O3	122.53 (7)
C2—C3—H3	119.9	O2—C8—C7	119.65 (7)
C4—C5—H5	120.2	O3—C8—C7	117.81 (7)
C4—C5—C6	119.55 (8)	C8—C7—H7A	109.5
C6—C5—H5	120.2	C8—C7—H7B	109.5
C3—C4—C5	120.66 (8)	C8—C7—H7C	109.5
C3—C4—H4	119.7	H7A—C7—H7B	109.5
C5—C4—H4	119.7	H7A—C7—H7C	109.5
O1—C2—C3	124.17 (7)	H7B—C7—H7C	109.5
			
O1—C2—C1—N1	−0.22 (10)	C4—C3—C2—O1	178.84 (8)
O1—C2—C1—C6	−178.85 (7)	C4—C3—C2—C1	−0.83 (12)
C3—C2—C1—N1	179.48 (7)	C4—C5—C6—C1	−0.39 (13)
C3—C2—C1—C6	0.85 (12)	C2—C3—C4—C5	0.22 (13)
C5—C6—C1—N1	−178.83 (7)	C6—C5—C4—C3	0.40 (14)
C5—C6—C1—C2	−0.24 (12)		

### Hydrogen-bond geometry (Å, º)

**Table:**

*D*—H···*A*	*D*—H	H···*A*	*D*···*A*	*D*—H···*A*
O1—H1···O3^i^	0.894 (14)	1.709 (14)	2.6025 (9)	177.8 (16)
N1—H1*A*···O2	0.930 (13)	1.807 (13)	2.7251 (9)	168.7 (12)
N1—H1*B*···O2^ii^	0.923 (12)	1.891 (12)	2.8019 (9)	168.8 (11)
N1—H1*C*···O3^iii^	0.935 (12)	1.834 (12)	2.7531 (8)	167.2 (12)
C6—H6···O3^iii^	0.95	2.55	3.2493 (11)	131

Symmetry codes: (i) *x*, *y*+1, *z*; (ii) −*x*, *y*+1/2, −*z*+3/2; (iii) *x*, −*y*+1/2, *z*+1/2.

## References

[bb1] Allen, F. H., Hoy, V. J., Howard, J. A. K., Thalladi, V. R., Desiraju, G. R., Wilson, C. C. & McIntyre, G. J. (1997). *J. Am. Chem. Soc.* **119**, 3477–3480.

[bb2] DeTitta, G. T. (1985). *J. Appl. Cryst.* **18**, 75–79.

[bb3] Dey, A., Desiraju, G. R., Mondal, R. & Howard, J. A. K. (2004). *Chem. Commun.* pp. 2528–2529. 10.1039/b407510b 15543263

[bb4] Dolomanov, O. V., Bourhis, L. J., Gildea, R. J., Howard, J. A. K. & Puschmann, H. (2009). *J. Appl. Cryst.* **42**, 339–341.

[bb5] Jagan, R. & Sivakumar, K. (2009). *Acta Cryst.* C**65**, o414–o418. 10.1107/S0108270109025724 19652327

[bb6] Jin, S. & Wang, D. (2013). *J. Mol. Struct.* **1037**, 242–255.

[bb7] Rigaku OD (2018). *CrysAlis PRO*. Rigaku Oxford Diffraction, Yarnton, England.

[bb8] Sheldrick, G. M. (2008). *Acta Cryst.* A**64**, 112–122. 10.1107/S0108767307043930 18156677

[bb9] Sheldrick, G. M. (2015). *Acta Cryst.* C**71**, 3–8.

[bb10] Smith, G., Wermuth, U. D., Healy, P. C. & White, J. M. (2011). *J. Chem. Crystallogr.* **41**, 1649–1662.

[bb11] Vivek, P. & Murugakoothan, P. (2014). *Appl. Phys. A*, **115**, 1139–1146.

[bb12] Yeşilel, O. Z., Paşaoğlub, H., Yılan, O. O. & Büyükgüngör, O. (2007). *Z. Naturforsch. Teil B*, **62**, 823–828.

[bb13] Zhao, Q. (2012). *Acta Cryst.* E**68**, o1535. 10.1107/S1600536812014973 PMC3344637 22590399

